# Vascular involvement in chronic thromboembolic pulmonary hypertension is associated with spirometry obstructive impairment

**DOI:** 10.1186/s12890-021-01779-x

**Published:** 2021-12-09

**Authors:** Asako Yanagisawa, Akira Naito, Takayuki Jujo-Sanada, Nobuhiro Tanabe, Keiichi Ishida, Goro Matsumiya, Rika Suda, Hajime Kasai, Ayumi Sekine, Toshihiko Sugiura, Ayako Shigeta, Seiichiro Sakao, Koichiro Tatsumi, Takuji Suzuki

**Affiliations:** 1 Department of Respirology, Graduate School of Medicine, Chiba University, 1-8-1 Inohana, Chuo-Ku, Chiba, 260-8670 Japan; 2 Pulmonary Hypertension Center, Chibaken Saiseikai Narashino Hospital, Narashino, 275-8580 Japan; 3 Department of Cardiovascular Surgery, Graduate School of Medicine, Chiba University, Chiba, 260-8670 Japan

**Keywords:** Chronic thromboembolic pulmonary hypertension, Obstructive ventilatory impairment, Respiratory impedance, CT angiography

## Abstract

**Background:**

Chronic thromboembolic pulmonary hypertension (CTEPH) is a type of pulmonary hypertension caused by persistent thromboembolism of the pulmonary arteries. In clinical practice, CTEPH patients often show obstructive ventilatory impairment, even in the absence of a smoking history. Recent reports imply a tendency for CTEPH patients to have a lower FEV_1.0_; however, the mechanism underlying obstructive impairment remains unknown.

**Methods:**

We retrospectively analyzed CTEPH patients who underwent a pulmonary function test and respiratory impedance test to evaluate their exertional dyspnea during admission for right heart catheterization from January 2000 to December 2019. We excluded patients with a smoking history to rule out the effect of smoking on obstructive impairment.

**Results:**

A total of 135 CTEPH patients were analyzed. The median FEV_1.0_/FVC was 76.0%, %FEV _1.0_ had a negative correlation with the mean pulmonary artery pressure and pulmonary vascular resistance and the CT Angiogram (CTA) obstruction score. A multivariate regression analysis revealed that the CTA obstruction score was an independent factor of a lower %FEV_1.0_. In the 54 patients who underwent pulmonary endarterectomy, %FEV_1.0_ was improved in some cases and was not in some. Mean PAP largely decreased after PEA in the better %FEV_1.0_ improved cases, suggesting that vascular involvement in CTEPH could be associated with spirometry obstructive impairment.

**Conclusion:**

%FEV_1.0_ had a significant correlation with the CTA obstruction score. Obstructive impairment might have an etiological relationship with vascular involvement. Further investigations could shed new light on the etiology of CTEPH.

**Supplementary Information:**

The online version contains supplementary material available at 10.1186/s12890-021-01779-x.

## Background

Chronic thromboembolic pulmonary hypertension (CTEPH) is a type of pulmonary hypertension caused by persistent thromboembolism of pulmonary arteries that fail to undergo complete thrombolysis after pulmonary thromboembolism (PTE) [1]. Multiple mechanisms are behind the occurrence of CTEPH. Several etiological factors, including infection, inflammation, and genetic susceptibility have been discussed as important pathogenetic factors [2–7], although some aspects remain unclear.

In clinical practice, CTEPH patients often show obstructive ventilatory impairment, even in the absence of a smoking history. A case report found that a CTEPH patient was misdiagnosed with bronchial asthma because of exertional dyspnea and a low FEV_1.0_ [8]. A recent report describing the pulmonary function test (PFT) findings of CTEPH patients showed that they tended to have a low FEV_1.0_ compared to healthy controls [9]. However, the relationship between the lung mechanics and hemodynamics is unclear.

In the present study, we hypothesized that some etiological component of CTEPH might affect the decrease in expiratory airflow. We evaluated the data of right heart catheterization (RHC), CT angiogram, respiratory function test of CTEPH patients.

## Methods

### Study population

The patients with CTEPH who underwent a PFT and respiratory impedance test to evaluate their exertional dyspnea during admission for RHC were enrolled from January 2000 to December 2019 at Chiba University Hospital. In this study the patients who underwent CT angiography for quantification of the pulmonary arterial thrombus obstruction were selected.

The patients who had a smoking history were excluded to rule out the effect of smoking on obstructive impairment. The criteria for the CTEPH diagnosis have been previously described [10]. CTEPH was defined as a mean pulmonary arterial pressure of 25 mmHg and pulmonary capillary wedge pressure of < 15 mmHg on RHC. Before the RHC diagnosis, patients received anticoagulation therapy for at least three months and underwent perfusion scintigraphy of the lung that showed a segmental blood flow distribution defect with no abnormality in the ventilation distribution.

### Right heart catheterization (RHC)

A 7.5-Fr Swan-Ganz catheter (Edwards Lifesciences, Irvine, CA, USA) was used for RHC. The pulmonary artery wedge pressure and pressure in the right atrium, right ventricle, main pulmonary artery, and right or left pulmonary artery were evaluated. The cardiac output (CO) was measured according to the thermodilution method. The procedure was described in greater detail in a previous study [11]. We conducted RHC testing during the same admission period as spirometry and the respiratory impedance test.

### Evaluation of sub-segmental pulmonary thrombi by CT angiography

Chronic thrombi within sub-segmental pulmonary arteries were quantified by enhanced CT scanning according to the modified methods of Qanadli [12], and the details were described previously [13]. Briefly; analyzing enhanced CT Angiogram (CTA) image of 0.5 mm thick, forty-two sub-segmental pulmonary arteries (twenty-two right-sided and twenty left-sided) were identified on each CT scan image and each sub-segmental artery was evaluated over the entire series of images. Each sub-segmental pulmonary artery was scored as follows: score 0: no thrombi; score 1: the artery was narrowed by chronic thrombi but contrast medium passed to distal areas; and score 2: the artery was obstructed by chronic thrombi and contrast medium did not pass to distal areas. The CTA obstruction score was defined as the total score of each sub-segmental score (maximum 84 point). Two investigators interpreted CT scan images in a blinded manner. To minimize bias, CTA obstruction scores were defined as the average value between the two investigators.

### Spirometry

The spirometry method is described previously [14, 15]; in brief, a PFT was performed for each patient according to the method described in the ATS/ERS guidelines [16]. The pulmonary function parameters, including the vital capacity (VC), forced VC (FVC), and forced expiratory volume in one second (FEV_1.0_), were measured by spirometry. The predicted values for VC, FVC, FEV_1.0_, ${\dot{\text{V}}}$50, ${\dot{\text{V}}}$25, maximum mid-expiratory flow (MMF), and peak expiratory flow (PEF) were determined based on reference values [17]. The total lung capacity and diffusion capacity for carbon monoxide (DLCO) were measured. DLCO was measured by the helium-dilution and single-breath methods, following the Japanese Respiratory Society guidelines [18].

### Respiratory impedance

Respiratory impedance was measured using a commercially available multi-frequency forced oscillation technique (FOT) device (Masterscreen IOS; Erich Jaeger, Hoechberg, Germany), following the standard recommendation [19]. The FOT measurements were performed before spirometry with the subjects in the sitting position with their neck in a comfortable neutral posture and wearing a nose clip, their cheeks firmly supported during measurement. The impedance against the oscillatory frequency was obtained. The measured respiratory system impedance was divided into the respiratory system resistance (Rrs) and respiratory system reactance (Xrs). Rrs is frequently interpreted as the airway caliber. The Rrs at 5 Hz (R5) and 20 Hz (R20) and the difference between the R5 and R20 (R5–R20) were recorded. Xrs reflects the elastic and inertial properties of the lung and thorax, Fres is the point at which Xrs = 0 is referred to as a resonant frequency, and ALX is the integral of reactance from X5 to Fres. These oscillatory indices were measured during the whole breath cycle.

### Statistical analyses

Wilcoxon’s signed-rank test, Fisher’s exact test, and a univariate regression analysis were used for comparisons between two parameters, where appropriate. The logistic regression model was used to calculate the adjusted odds ratio (OR) with the 95% confidence interval (CI) for obstructive impairment associated with hemodynamics in CTEPH. A multivariate logistic regression analysis was performed to evaluate the factors associated with the decreased expiratory airflow.

The results are presented as the mean ± SEM or median with interquartile range (IQR). *p* values < 0.05 were considered statistically significant. All statistical analyses were performed using a commercially available software program (JMP 9.0.2, Japanese version; SAS Institute Inc., Tokyo, Japan).

## Result

### Patients’ characteristics

The clinical characteristics and respiratory function of CTEPH patients are summarized in Tables 1 and 2. A total of 251 patients were diagnosed with CTEPH from 2000 to 2019, and the 90 patients with a smoking history were excluded as mentioned above. Finally, we analyzed 135 patients who had all dataset of PFT, RHC, and CT angiography.

**Table 1:** Baseline characteristics of the patients with CTEPH

	CTEPH (n = 135)
Age (years)	64 (52–70)
Male/female, n	27/108
Body weight (kg)	52.9 (48.0–60.4)
BMI (kg/m^2^)	21.6 (20.0–23.8)
WHO-FC (I: II: III: IV)	10:74:50:1
Type (occult/recurrent)	80/55
Hb (g/dL)	13.8 (12.6–14.8)
BNP (pg/mL)	86.3 (29.2–288)
CRP (g/dL)	0.1 (0.1–0.2)
ATIII (%)	99 (89.8–109)
Systolic blood pressure (mmHg)	124 (110–142)
Diastolic blood pressure (mmHg)	73 (63–82)
mPAP (mmHg)	43 (35–51)
PVR (dyne sec cm^−5^)	689 (482–983)
RAP (mmHg)	8.0 (6.0–11.5)
CO (L/min)	3.90 (3.37–4.61)
CI (L/min/m^2^)	2.53 (2.18–2.95)
PaO_2_ (mmHg)	56 (51.8–65.9)
PaCO_2_ (mmHg)	37.7 (34.3–42.7)
PvO_2_ (mmHg)	33.8 (31.1–35.9)
SvO_2_ (%)	62.7 (58.5–68.5)
AaDO_2_ (mmHg)	48.0 (39.9–53.4)
6MWD (m)	369 (306–424)
CTA obstruction score	26 (18–34)

Values are expressed as the median with the interquartile range

BMI, body mass index; WHO-FC, WHO-functional class; Hb, hemoglobin; BNP, brain natriuretic peptide; CRP, C-reactive protein; ATIII, antithrombin III; mPAP, mean pulmonary arterial pressure; PVR, pulmonary vascular resistance; CO, cardiac output; CI, cardiac index; PaO_2_, partial pressure of arterial oxygen; PaCO_2_, partial pressure of arterial carbon dioxide; PvO_2_, mixed venous oxygen pressure; SvO_2_, mixed venous oxygen saturation; AaDO_2_, alveolar-arterial oxygen difference; 6MWD, 6-minute walk distance; CTA, CT angiogram

**Table 2:** Respiratory function indices in patients with CTEPH

	Measured values	Expected values	*p* value
VC (L)	2.55 (2.05–3.07)	2.57 (2.33–2.92)	< 0.001**
%VC	94.8 (83.1–101.7)		
FVC (L)	2.55 (1.98–3.00)	2.48 (2.26–2.83)	< 0.001**
%FVC	94.6 (84.2–105.1)		
FEV_1.0_ (L)	1.91 (1.47–2.32)	2.04 (1.82–2,46)	< 0.001**
FEV_1.0_/FVC (%)	76.0 (70.0–82.1)	81.9 (79.3–85.9)	< 0.001**
%FEV_1.0_	83.6 (73.8–98.3)		
MMF (L)	1.40 (0.87–2.17)	2.60 (2.30–3.10)	< 0.001**
%MMF	48.9 (35.1–71.6)		
${\dot{\text{V}}}$50	2.02 (1.28–2.75)	2.95 (2.66–3.5)	< 0.001**
%${\dot{\text{V}}}$50	59.9 (42.9–82.6)		
${\dot{\text{V}}}$25 (L)	0.47 (0.29–0.82)	1.06 (0.87–1.39)	< 0.001**
%${\dot{\text{V}}}$25	41.8 (29.0–58.5)		
PEF (L/s)	5.54 (4.42–6.67)	5.62 (5.37–6.08)	< 0.001**
> 5% of ATI, n (%)	24 (17.8%)		
Restrictive ventilatory impairment, n (%)	22 (16.3%)		
Obstructive ventilatory impairment, n (%)	34 (25.2%)		
Mixed ventilatory impairment, n (%)	3 (2.2%)		
DLco (mL/min/mmHg)	13.1 (10.9–16.0)	17.0 (15.1–20.1)	< 0.001**
%DLco	77.0 (64.8–86.5)		
TLC (L)	4.77 (3.92–5.64)	4.44 (4.11–4.97)	N.S
RV (L)	1.86 (1.58–2.24)	1.82 (1.69–1.96)	N.S
RV/TLC (%)	39.9 (35.4–43.9)		

Values are expressed as the median with the interquartile range

VC, vital capacity; FEV_1.0_, forced expiratory volume in 1 second; FVC, forced vital capacity; MMF, maximal mid‒expiratory flow; ${\dot{\text{V}}}$50 and ${\dot{\text{V}}}$25, flow at 50,and 25% of vital capacity; PEF, peak expiratory flow; ATI, air trapping index; DLco, diffused capacity of carbon monoxide; TLC, total lung capacity; RV, residual volume

**p < 0.01

Female patients outnumbered male patients (108: 27), and 41% of all patients had acute pulmonary embolism episode (Table 1). None of the patients enrolled in this study were on bronchodilator treatment.

### Spirometry and respiratory impedance test findings

Obstructive ventilatory impairment was observed in 34/135 (25.2%) with a cut-off value of FEV_1.0_/FVC (%) of 70%, and the overall median FEV_1.0_/FVC decreased to 76.0%, which is close to the lower limit. The MMF, ${\dot{\text{V}}}$50, and ${\dot{\text{V}}}$25 V, which were effort-independent components during the end-expiratory portion of the flow-volume curve, were lower than their expected values. These results suggested the existence of peripheral airway obstruction, although there was no significant difference in the residual volume or total lung capacity (Table 2). Regarding the relationship between the spirometry and respiratory impedance test results, no correlation was noted between the %FEV_1.0_/ %MMF and R5, R20, or R5-R20 (Additional file 1).

### Correlation between the PFT and pulmonary hemodynamic parameters

**Fig. 1:**
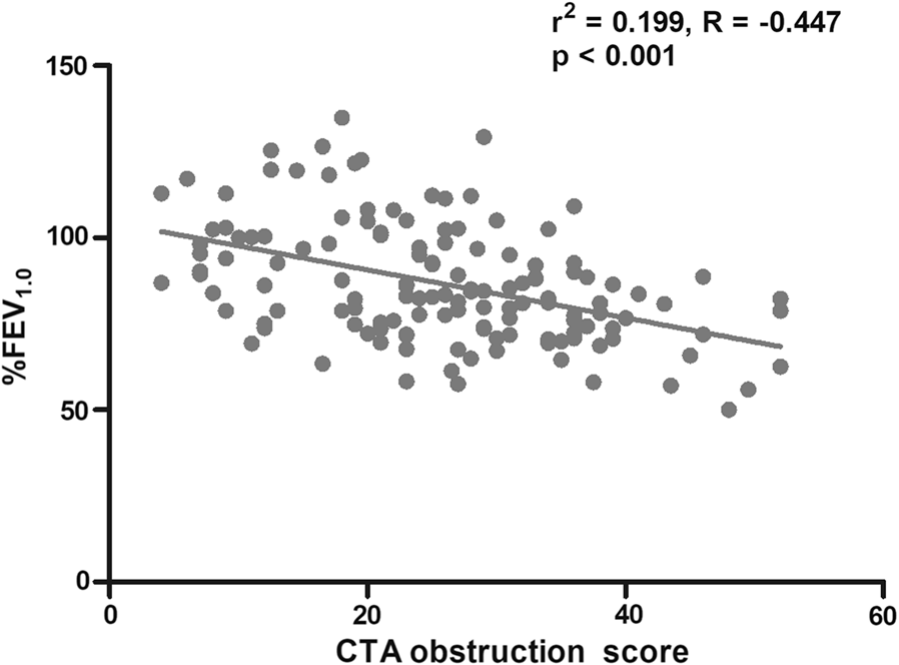
Correlation between CTA obstruction score and %FEV_1.0_. The CT angiogram obstruction score was defined as the total score of each sub-segmental pulmonary arteries (score 0: no thrombi; score 1: the artery was narrowed by chronic thrombi but contrast medium passed to distal areas; score 2: the artery was obstructed by chronic thrombi and contrast medium did not pass to distal areas). Significant correlation was observed between CTA obstruction score and the %FEV_1.0_

We analyzed the correlation between the PFT parameters and the pulmonary hemodynamics (mPAP, PVR, cardiac index) / CTA obstruction score. Among the PFT parameters shown in Table 2, the %FEV_1.0_ was inversely correlated with mPAP, PVR and CTA obstruction score, and positively correlated with the cardiac index (Table 3, Fig. 1).

**Table 3:** The correlation between PFT parameters and pulmonary hemodynamic parameters, CTA score at initial diagnosis

	%FEV_1.0_		%MMF		%${\dot{\text{V}}}$50		%${\dot{\text{V}}}$25	
	r	*p*	r	*p*	r	*p*	r	*p*
mPAP	− 0.266	0.002*	− 0.204	0.0177*	− 0.271	0.0015*	− 0.194	0.0244*
PVR	− 0.284	0.0009**	− 0.223	0.0096**	− 0.241	0.0051	− 0.195	0.0245*
CO	0.127	0.1408	0.176	0.0410*	0.137	0.113	0.152	0.0797
CI	0.219	0.0109*	0.216	0.0121*	− 0.186	0.0306*	0.229	0.0078**
CTA	− 0.445	< 0.0001	− 0.203	0.0181*	− 0.256	0.0028*	− 0.132	0.127

PFT, pulmonary function test; mPAP, mean pulmonary arterial pressure; PVR, pulmonary vascular resistance; CO, cardiac output; CI, cardiac index; CTA, CTA obstruction score; FEV_1.0_, forced expiratory volume in 1 second; FVC, forced vital capacity; MMF, maximal mid‒expiratory flow; ${\dot{\text{V}}}$50 and ${\dot{\text{V}}}$25: flow at 50,and 25% of vital capacity

^*^p < 0.05; **p < 0.01

The respiratory impedance parameters were poorly correlated with the pulmonary hemodynamic parameters, while the R20 and X5 had mild correlation with the cardiac indices (Additional file 2).

When patients were divided into two groups according to the median %FEV_1.0_ (83.6%), the mPAP, PVR, RAP, CRP, BNP, and CTA obstruction score values were significantly higher, while the cardiac index and systolic BP values were lower in the lower %FEV_1.0_ group than in the higher %FEV_1.0_ group (Table 4).

**Table 4:** Baseline characteristics and respiratory function indices subdivided into groups according to median %FEV_1.0_

	%FEV_1.0_ < 83.6%	%FEV_1.0_ ≧ 83.6%	*p* value
Age (years)	58.7 ± 1.7	62.2 ± 1.4	N.S
Male/female, n	19/48	8/60	0.0148*
Body weight (kg)	57.2 ± 1.5	52.8 ± 1.05	0.0339*
BMI (kg/m^2^)	22.4 ± 0.5	21.8 ± 0.3	N.S
WHO-FC (I: II: III: IV)	6/34/26/1	4/40/24/0	N.S
Type (Occult/Recurrent)	40/27	40/28	N.S
Hb (g/dL)	13.9 ± 0.2	13.6 ± 0.2	N.S
BNP (pg/mL)	296 ± 51	162 ± 27	0.0098**
CRP (g/dL)	0.27 ± 0.06	0.21 ± 0.05	0.0173*
ATIII (%)	97.4 ± 1.8	100.3 ± 1.6	N.S
Systolic SBP	123 ± 3	129 ± 2	0.0346*
Diastolic SBP	73 ± 2	74 ± 2	N.S
mPAP (mmHg)	45.1 ± 1.3	41.1 ± 1.2	0.0331*
PVR (dyne sec cm^−5^)	815 ± 45	689 ± 34	0.0334*
RAP (mmHg)	10.1 ± 0.53	7.65 ± 0.43	0.0008**
CO (L/min)	4.05 ± 0.14	4.23 ± 0.13	N.S
CI (L/min/m^2^)	2.51 ± 0.08	2.77 ± 0.09	0.0307*
CTA obstruction score	30.1 ± 1.3	21.6 ± 1.2	< 0.0001**

Values are expressed as the mean ± standard error of the mean

BMI, body mass index; WHO-FC, WHO-functional class; Hb, hemoglobin; BNP, brain natriuretic peptide; CRP, C-reactive protein; AT III, antithrombin III, SBP, systemic blood pressure; PAP, pulmonary arterial pressure; PVR, pulmonary vascular resistance; CO, cardiac output; CI, cardiac index

*p < 0.05; **p < 0.01

### Logistic regression analyses of low %FEV_1.0_

Given the negative correlation noted between pulmonary arterial obstruction or pulmonary hemodynamics and the %FEV_1.0_, we tried to identify the predictors of a low %FEV_1.0_. Dividing the patients into two groups according to the median %FEV_1.0_ (83.6%) as in Table 4, a univariate logistic regression analysis showed that higher BW, higher BNP, higher mPAP, higher PVR, lower CI, higher RAP, higher CTA obstruction score were associated with a low %FEV_1.0_ in CTEPH patients. A multivariate logistic regression analysis showed that higher CTA obstruction score was independent factors associated with a low %FEV_1.0_ (Table 5).

**Table 5:** Predictors of low %FEV_1.0_ according to a multivariate logistic regression analysis

Predictors of low %FEV_1.0_						
	Univariate			Multivariate		
	Odds ratio	95% CI	*p* value	Odds ratio	95% CI	*p* value
BW	1.041	1.006–1.077	0.0170*	1.031	0.981–1.084	0.214
BNP	1.001	1.000–1.003	0.0164*	1.000	0.999–1.002	0.809
CRP	1.369	0.596–3.144	0.443			
sBP	0.984	0.967–1.001	0.0627			
mPAP	1.041	1.005–1.078	0.020*	1.073	0.980–1.174	0.124
PVR	1.001	1.000–1.002	0.0267*	0.998	0.994–1.002	0.267
CI	0.560	0.329–0.952	0.0266*	0.369	0.119–1.143	0.076
RAP	1.182	1.071–1.305	0.0003**	1.071	0.951–1.208	0.256
CTA	1.084	1.044–1.126	< 0.0001**	1.074	1.031–1.120	< 0.001**

^*^p < 0.05; **p < 0.01

BW, body weight; BNP, brain natriuretic peptide; CRP, C-reactive protein; sBP, systolic systemic blood pressure; PAP, pulmonary arterial pressure; PVR, pulmonary vascular resistance; RAP, right atrium pressure; CTA, CTA obstruction score

### Effect of pulmonary endarterectomy (PEA) on the PFT

We analyzed the effect of PEA on the PFT to further assess the correlation between respiratory function and the pulmonary hemodynamics. The pulmonary hemodynamics and pulmonary function one year after PEA are reported in Additional file 3.

**Fig. 2:**
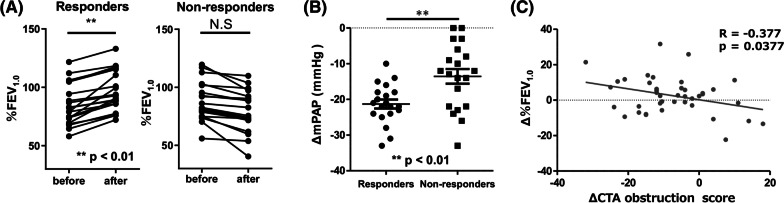
**A** Change in %FEV_1.0_ of responders and non-responders group. **B** Effect of PEA on improvement of mPAP (ΔmPAP) between responders and non-responders_._ **C** The correlation between ΔCTA obstruction score and Δ%FEV_1.0_. The largest 20 cases regarding the value of %FEV_1.0_ positive and negative difference before and after PEA was defined as responder and non-responder, respectively. **A** The difference in value was named as Δ%FEV_1.0_. **B** Mean PAP was better improved in the responders group compared to the non-responders group. **C** In 29 patients in whose CTA obstruction score could be assessed postoperatively, ΔCTA obstruction score and Δ%FEV_1.0_ were negatively correlated

To examine whether it is relevant between vascular involvement and obstructive impairment change after PEA, responder and non-responder in %FEV_1.0_ were extracted from the 54 patients receiving PEA. The largest 20 cases regarding the value of %FEV_1.0_ positive and negative difference before and after PEA was defined as responder and non-responder, respectively, and the difference in value was named as Δ%FEV_1.0_ (Fig. 2A). Mean PAP was better improved in the responders group compared to the non-responders (Fig. 2B). In 29 patients in whose CTA obstruction score could be assessed postoperatively, ΔCTA obstruction score and Δ%FEV_1.0_ were negatively correlated (Fig. 2C).

## Discussion

In the present study we analyzed 135 CTEPH patients and found that a decreased %FEV_1.0_ and %MMF were correlated with the degree of vascular obstruction and pulmonary hemodynamics. On analyzing the CTA obstruction score, pulmonary hemodynamics and PFT parameters one year after PEA, the %FEV_1.0_ was found to be improved with the improvement of CTA obstruction score and pulmonary hemodynamics. The improvement of the %FEV_1.0_ due to the improvement of vascular occlusion or stenosis is consistent with the findings of a previous study by Takei et al. describing balloon pulmonary angioplasty cases [20]. Those findings suggest that the obstructive impairment in CTEPH patients might have an etiological correlation with the degree of vascular obstruction.

The present findings newly suggest that airway obstruction has an etiological correlation with vascular involvement in CTEPH. What we consider most important is that %FEV_1.0_ was increased with improvement in vascular involvement after PEA. Besides, we excluded the CTEPH patients who had a smoking history, which is well-known cause of obstructive impairment. It could be that the obstructive impairment results from vascular involvement.

In a previous case report, a CTEPH patient was misdiagnosed with bronchial asthma because of exertional dyspnea and a low FEV_1.0_ [8]. A low FEV_1.0_ does not necessarily mean the presence of airway disease such as COPD and asthma in patients having exertional dyspnea. We should keep the possibilities of the pathophysiological condition with right heart burden including CTEPH in mind while carrying out a physical examination, chest X-ray, electro-cardiogram, etc.

Summarizing the previous studies, the pathological changes in CTEPH are characterized by thrombotic occlusion and remodeling of non-obstructed arteries induced by the flow diversion from obstructed arteries [21, 22]. In the area where pulmonary arteries are occluded, peripheral opacites caused by infarction are frequent findings on chest CT [23, 24]. Lung infarction could affect local airflow by collapsing the alveolar- bronchiole regions. On the other side, many papers described that the disparity in segmental vessel size reflecting the irregular distribution of emboli within the lungs was a characteristic finding of the CTEPH [23, 25, 26]. Local enlargement of the peripheral pulmonary arteries may lead to compression of bronchi nearby.

Another possible important factor of flow limitation underlying CTEPH is the concept of ‘‘inflammatory thrombosis’’. Various inflammatory substances are reportedly generated from blood clots and remodeled pulmonary arteries, including CRP [27], TNF-α [28], and MCP-1 [29]. These cytokines are also reported to enhance the bronchial contraction [30–32]. In our results, significant correlation was observed between CTA obstruction score and the %FEV_1.0_ (Fig. 1). Inflammatory substances from injured endothelium, inflammatory cells and organizing thrombus in the subsegmental pulmonary arteries might lead to contraction of bronchioles nearby. As one candidate cytokines, we measured the plasma TNF-α levels in a part of subjects as an exploratory research, however, no significant correlation was observed between plasma TNF-α level and %FEV_1.0_ / CTA score (Additional file 4). Comprehensive circulating or the local cytokine around the thrombi could not be evaluated in this study. Thus, the association between the obstructive impairment and the cytokine levels remains a future challenge.

This is the first report to evaluate the respiratory impedance in patients with CTEPH, although the respiratory impedance parameters were poorly correlated with the spirometry parameters. The FOT is a method of measuring the lung mechanics that is better at the early detection of flow limitation than spirometry [33–35]. The correlation between the FOT and spirometry parameters has been previously reported. In COPD and asthma, R5 moderately correlates with the FEV_1.0_ and is thus used as an index of airway obstruction [36–38]. X5, Fres, and ALX are indicators of resistance of lung expansion or shrinkage and reflect abnormalities of the lung parenchyma [37]. In the present study, the FOT parameters were poorly correlated with spirometry, in contrast to the previous studies mentioned above (Additional file 1). Because the respiratory impedance in CTEPH patients showed differing trends from those in COPD or asthma [37, 38], there might be an etiological difference between the obstructive impairment seen in CTEPH and that in COPD/asthma. Further investigations will help clarify the entire CTEPH etiology.

Several limitations associated with the present study warrant mention. First, the study design was a retrospective, single-center, observational study. Second, we were unable to clarify the detailed mechanism underlying the respiratory function changes because we were unable to perform pathological analyses. An additional multicenter investigation is therefore required.

## Conclusion

In CTEPH patients without a smoking history, the %FEV_1.0_ showed a significant correlation with the pulmonary arterial obstruction and the pulmonary hemodynamics. Obstructive impairment might have an etiological relationship with vascular involvement. A further investigation focusing on the obstructive impairment in CTEPH patients may shed new light on the CTEPH etiology.

## Supplementary Information

**Additional file 1.** Correlation of representative spirometry and respiratory impedance parameters in CTEPH patients. *p < 0.05; **p < 0.01. FEV_1.0_, forced expiratory volume in 1 second; FVC, forced vital capacity; MMF, maximal mid‒expiratory flow

**Additional file 2.** Correlation of pulmonary hemodynamics with respiratory impedance parameters in CTEPH patients. *p < 0.05. mPAP, mean pulmonary arterial pressure; PVR, pulmonary vascular resistance; CO, cardiac output; CI, cardiac index

**Additional file 3.** Effects of pulmonary endarterectomy on the respiratory function. *p < 0.05; **p < 0.01. Values are expressed as the mean ± standard error of the mean. VC, vital capacity; FVC, forced vital capacity; FEV_1.0_, forced expiratory volume in 1 second; TLC, total lung capacity; PaO_2_ partial pressure of arterial oxygen; PaCO_2_, partial pressure of arterial carbon dioxide; SvO_2_, mixed venous oxygen saturation; PvO_2_, mixed venous oxygen pressure

**Additional file 4.** Correlation of plasm level of TNF-α with %FEV_1.0_ (A) and CTA obstruction score (B) in CTEPH patients. TNF-α; tumor necrosis factor

## Data Availability

The datasets used and/or analyzed during the current study are available from the corresponding author on reasonable request.

## Abbreviations

CTEPH: Chronic thromboembolic pulmonary hypertension
RHC: Right heart catheterization
mPAP: Mean pulmonary artery pressure
PVR: Pulmonary vascular resistance
CO: Cardiac output
CI: Cardiac index
CTA: CT Angiogram
PFT: Pulmonary function test
VC: Vital capacity
FVC: Forced vital capacity
FEV_1.0_: Forced expiratory volume in one second
MMF: Maximum mid-expiratory flow
PEF: Peak expiratory flow
DLCO: Diffusion capacity for carbon monoxide
FOT: Multi-frequency forced oscillation technique
Rrs: Respiratory system resistance
Xrs: Respiratory system reactance
R5: Respiratory system resistance at 5 Hz
R20: Respiratory system resistance at 20 Hz
R5–R20: The difference between the R5 and R20
PEA: Pulmonary endarterectomy
