# Supplementary material for: Vascular involvement in chronic thromboembolic pulmonary hypertension is associated with spirometry obstructive impairment
Source: BMC Pulm Med. 2021 Dec 9;21:407. doi: 10.1186/s12890-021-01779-x (PMC8656012; doi:10.1186/s12890-021-01779-x)
Supplement: Supplementary file 1 — Additional file 1. Correlation of representative spirometry and respiratory impedance parameters in CTEPH patients. *p < 0.05; **p < 0.01. FEV1.0, forced expiratory volume in 1 second; FVC, forced vital capacity; MMF, maximal mid‒expiratory flow [file 12890_2021_1779_MOESM1_ESM.docx]

**Additional file 1.** Correlation of representative spirometry and respiratory impedance parameters in patients with CTEPH

|  | **FEV_1.0_/FVC** | **%FEV_1.0_** | **%MMF** |
| --- | --- | --- | --- |
| **R5** | -0.104 | -0.113 | -0.139 |
| **R20** | -0.069 | -0.078 | -0.101 |
| **R5-R20** | -0.126 | -0.145 | -0.164 |
| **X5** | -0.027 | 0.256^*^ | 0.145 |
| **Fres** | 0.0400 | -0.109 | -0.039 |
| **ALX** | -0.041 | -0.257^*^ | -0.183 |

^*^*p* < 0.05; ^**^*p <* 0.01. FEV_1.0_, forced expiratory volume in 1 second; FVC, forced vital capacity; MMF, maximal mid‒expiratory flow
