# Supplementary material for: Vascular involvement in chronic thromboembolic pulmonary hypertension is associated with spirometry obstructive impairment
Source: BMC Pulm Med. 2021 Dec 9;21:407. doi: 10.1186/s12890-021-01779-x (PMC8656012; doi:10.1186/s12890-021-01779-x)
Supplement: Supplementary file 2 — Additional file 2. Correlation of pulmonary hemodynamics with respiratory impedance parameters in CTEPH patients. *p < 0.05. mPAP, mean pulmonary arterial pressure; PVR, pulmonary vascular resistance; CO, cardiac output; CI, cardiac index [file 12890_2021_1779_MOESM2_ESM.docx]

**Additional file 2.** Correlation of pulmonary hemodynamics with respiratory impedance parameters in CTEPH patients

|  | **mPAP** | **PVR** | **CO** | **CI** |
| --- | --- | --- | --- | --- |
| **R5** | 0.150 | 0.056 | 0.150 | 0.226 |
| **R20** | 0.215 | 0.084 | 0.194 | 0.287^*^ |
| **R5-R20** | 0.022 | 0.044 | -0.010 | 0.007 |
| **X5** | -0.223 | -0.130 | -0.160 | -0.274^*^ |
| **Fres** | -0.039 | 0.003 | 0.011 | 0.068 |
| **ALX** | 0.071 | -0.006 | 0.161 | 0.206 |

^*^*p* < 0.05. mPAP, mean pulmonary arterial pressure; PVR, pulmonary vascular resistance; CO, cardiac output; CI, cardiac index
