# Supplementary material for: Vascular involvement in chronic thromboembolic pulmonary hypertension is associated with spirometry obstructive impairment
Source: BMC Pulm Med. 2021 Dec 9;21:407. doi: 10.1186/s12890-021-01779-x (PMC8656012; doi:10.1186/s12890-021-01779-x)
Supplement: Supplementary file 3 — Additional file 3. Effects of pulmonary endarterectomy on the respiratory function. *p < 0.05; **p < 0.01. Values are expressed as the mean ± standard error of the mean. VC, vital capacity; FVC, forced vital capacity; FEV1.0, forced expiratory volume in 1 second; TLC, total lung capacity; PaO2 partial pressure of arterial oxygen; PaCO2, partial pressure of arterial carbon dioxide; SvO2, mixed venous oxygen saturation; PvO2, mixed venous oxygen pressure [file 12890_2021_1779_MOESM3_ESM.docx]

**Additional file 3.** Effects of pulmonary endarterectomy on the respiratory function

|  | **Before PEA** | **1 year after PEA** | **p-value** |
| --- | --- | --- | --- |
| VC (L) | 2.67 ± 0.10 | 2.68 ± 0.10 | N.S |
| FVC (L) | 2.68 ± 0.10 | 2.61 ± 0.11 | N.S |
| ％FVC | 95.5 ± 2.02 | 95.4 ± 2.23 | N.S |
| FEV_1.0_ (L) | 2.00 ± 0.08 | 1.99 ± 0.08 | N.S |
| ％FEV_1.0_ | 87.3 ± 2.3 | 89.8 ± 2.4 | 0.025^*^ |
| FEV_1.0_/FVC (%) | 75.0 ± 1.2 | 75.2 ± 1.1 | N.S |
| ％TLC | 103.8 ± 2.15 | 101.7 ± 1.95 | N.S |
| mPAP (mmHg) | 43.5 ± 1.2 | 25.8 ± 1.0 | < 0.001^**^ |
| PVR (dyne･sec･cm^-5^) | 730 ± 35 | 342 ± 25 | < 0.001^**^ |
| CO (L/min) | 3.99 ± 0.11 | 4.31 ± 0.10 | 0.005^**^ |
| CI (L/min/m^2^) | 2.53 ± 0.06 | 2.69 ± 0.05 | 0.029^*^ |
| RAP (mmHg) | 8.20 ± 0.46 | 4.30 ± 0.31 | < 0.001^**^ |
| PaO_2_ (mmHg) | 59.3 ± 1.56 | 70.4 ± 2.03 | < 0.001^**^ |
| PaCO_2_ (mmHg) | 36.9 ± 0.5 | 40.3 ± 0.5 | < 0.001^**^ |
| SvO_2_ (%) | 62.1 ± 0.8 | 66.8 ± 0.8 | < 0.001^**^ |
| PvO_2_ (mmHg) | 33.9 ± 0.4 | 40.3 ± 0.5 | < 0.001^**^ |

Values are expressed as the mean ± standard error of the mean. ^*^*p* < 0.05; ^**^*p* < 0.01. VC, vital capacity; FVC, forced vital capacity; FEV_1.0_, forced expiratory volume in 1 second; TLC, total lung capacity; PaO_2_ partial pressure of arterial oxygen; PaCO_2_, partial pressure of arterial carbon dioxide; SvO_2_, mixed venous oxygen saturation; PvO_2_, mixed venous oxygen pressure
