# Supplementary figures and images for: Vascular involvement in chronic thromboembolic pulmonary hypertension is associated with spirometry obstructive impairment
Source: BMC Pulm Med. 2021 Dec 9;21:407. doi: 10.1186/s12890-021-01779-x (PMC8656012; doi:10.1186/s12890-021-01779-x)

## Slide 1
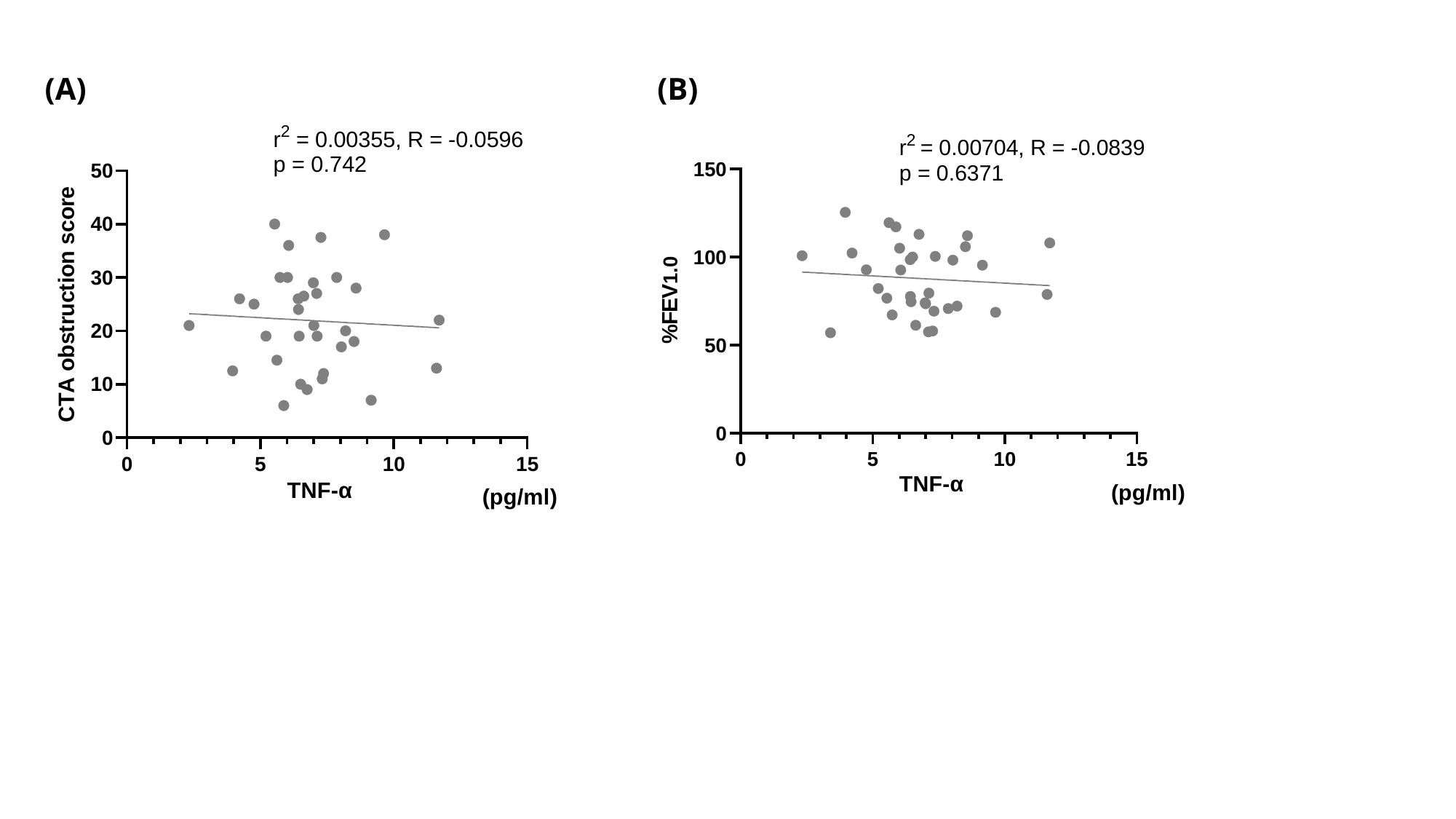

(A)
(B)

Supplement: Supplementary file 4 — Additional file 4. Correlation of plasm level of TNF-α with %FEV1.0 (A) and CTA obstruction score (B) in CTEPH patients. TNF-α; tumor necrosis factor [file 12890_2021_1779_MOESM4_ESM.pptx]
